# In Situ X-ray Absorption Spectroscopy of LaFeO_3_ and LaFeO_3_/LaNiO_3_ Thin Films in the Electrocatalytic Oxygen Evolution Reaction

**DOI:** 10.1021/acs.jpcc.3c07864

**Published:** 2024-03-20

**Authors:** Qijun Che, Iris C. G. van den Bosch, Phu T. P. Le, Masoud Lazemi, Emma van der Minne, Yorick A. Birkhölzer, Moritz Nunnenkamp, Matt L. J. Peerlings, Olga V. Safonova, Maarten Nachtegaal, Gertjan Koster, Christoph Baeumer, Petra de Jongh, Frank M. F. de Groot

**Affiliations:** † Materials Chemistry and Catalysis, Debye Institute for Nanomaterials Science, Utrecht University, Universiteitsweg 99, Utrecht 3584 CG, The Netherlands; ‡ MESA+ Institute for Nanotechnology, University of Twente, Enschede 7500 AE, The Netherlands; § PSI, Villigen CH-5232, Switzerland

## Abstract

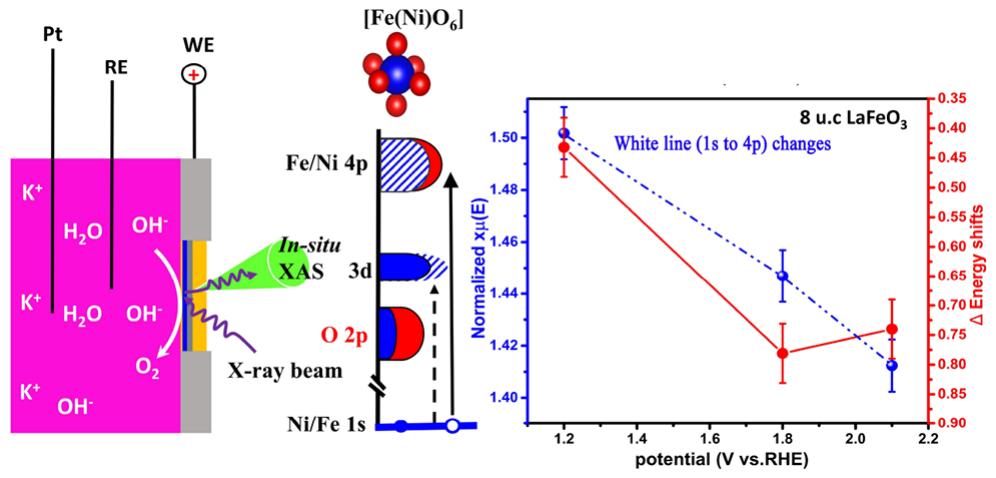

We study the electrocatalytic oxygen evolution reaction using in situ X-ray absorption spectroscopy (XAS) to track the dynamics of the valence state and the covalence of the metal ions of LaFeO_3_ and LaFeO_3_/LaNiO_3_ thin films. The active materials are 8 unit cells grown epitaxially on 100 nm conductive La_0.67_Sr_0.33_MnO_3_ layers using pulsed laser deposition (PLD). The perovskite layers are supported on monolayer Ca_2_Nb_3_O_10_ nanosheet-buffered 100 nm SiN_*x*_ membranes. The in situ Fe and Ni K-edges XAS spectra were measured from the backside of the SiN_*x*_ membrane using fluorescence yield detection under electrocatalytic reaction conditions. The XAS spectra show significant spectral changes, which indicate that (1) the metal (co)valencies increase, and (2) the number of 3d electrons remains constant with applied potential. We find that the whole 8 unit cells react to the potential changes, including the buried LaNiO_3_ film.

## Introduction

The electrocatalytic oxygen evolution reaction (OER) via water electrolysis needs large overpotentials, which is regarded as a bottleneck in developing a hydrogen economy.^1,2^ Benchmark Ir/Ru-oxide-based OER electrocatalysts have been significantly obstructed from widespread practical viability in terms of cost and scarcity.^3^ Transition metal oxides, as promising earth-abundant alternatives, have outperformed noble-metal-based electrocatalysts in alkaline electrolytes and aroused great interest due to their tunable properties in geometry and electronic structures.^4−7^ The e_g_ symmetry electronic states of the transition metal sites were proposed as a descriptor to predict OER activity trends,^8^ which was contradicted in other experiments.^9^ Nevertheless, fully capturing the population of the e_g_ states is a tremendous challenge. For example, LaCoO_3_ is sensitive to temperature and strain, inducing spin transitions that mix low-spin, high-spin, and intermediate-spin states.^6^ We further note that the geometric and electronic structure properties of the surface are different from the bulk.^10,11^ The electrocatalytic oxygen evolution at the electrode/electrolyte interface is caused by lattice oxygen participation mechanisms^12^ and surface reconstructions.^13−15^ Identification of the reconstructed layer is complex due to an amorphization of the surface layer.^16−18^ The electronic structure under operating conditions is highly debated, for example, the possibility of high valences of iron, cobalt, and nickel. Friebel et al. suggested that Fe^3+^ in Ni_1–*x*_Fe_*x*_OOH acts as an active site, based on density functional theory (DFT) calculations;^19^ Al Samarai et al. proposed that the γ-NiOOH phase is active in the evolution of Ni_3_MnO_4_ by the in situ 2p3d resonant inelastic X-ray scattering (RIXS);^20^ and Chen et al. detected the Fe^4+^ species by using in situ Mössbauer spectroscopy in nickel–iron hydroxide.^21^

In this study, we will use oxide thin fims, i.e., 8 unit cell LaFeO_3_ on 100 nm conductive La_0.67_Sr_0.33_MnO_3_ supported on monolayer Ca_2_Nb_3_O_10_ nanosheet-buffered 100 nm SiN_*x*_ membranes. In a second sample, we add 8 unit cell LaNiO_3_ under the LaFeO_3_ layer. Oxide thin fims have been used for the electrocatalysis reactions; Weber and Gunkel recently have summarized the strategy of epitaxial thin film catalysts and their potential importance to OER.^22^ A review on oxide thin fims for the OER activity trends has recently been published by Antipin and Risch.^23^ For example, Stoerzinger et al. studied thin films of La(Ca,Sr)MnO_3_ on SrTiO_3_ for the oxygen reduction reaction (ORR),^24^ and Heymann et al. studied thin bilayers of La(Sr)CoO_3_ systems for the OER reaction, suggesting that bilayer films exhibited hole accumulation at the surface.^25^ The bilayer thin films in many cases exhibit special properties and play an important role in direct or indirect activity, in which they are involved in the surface and subsurface. Akbashev et al. reported SrTiO_3_ with sublayer SrRuO_3_ catalyst, in which the subsurface of SrRuO_3_ efficiently activates the top layer of SrTiO_3._^26^ Regarding the few unit cell thin films of LaFeO_3_ and LaFeO_3_/LaNiO_3_ system, we regard the near surface region as relevant for OER activity, in agreement with Vitale-Sullivan’s view.^27^

In the investigation of complex transition-metal systems, in situ X-ray absorption spectroscopy (XAS) plays an important role in probing the electronic structure (valence, spin states, covalence, and charge transfer) under working conditions regardless of long-range or short-range order.^28^ We used fluorescence yield (FY) XAS, which is a bulk-sensitive technique. In order to reveal the properties of the near-surface layer, we used very thin films. The high crystal quality, single out-of-plane crystal orientation of these thin films, and accessibility for in situ XAS are ensured via an epitaxial growth on an X-ray transparent membrane. Using a membrane enables FY detection from the backside, which is advantageous compared to measuring through the electrolyte, which strongly absorbs X-rays.

Single-crystal LaNiO_3_ and LaFeO_3_ perovskites have the following properties: 1) the Ni and Fe ions have a formal valence of 3+, and the La^3+^ ion has a [Xe] configuration without any partly filled orbitals; 2) LaNiO_3_ is metallic at all temperatures, and Ni^3+^ is 3d^7^ of mainly low spin (t_2g_^6^e_g_^1^) in O_h_ symmetry.^29,30^ The ground state of LaNiO_3_ is mixed with some high-spin character due to the competition between crystal field, spin–orbit coupling, and electrostatic interactions; 3) LaFeO_3_ is an antiferromagnetic insulator (Néel temperature, *T*_N_ ≈ 740 K),^31^ where Fe^3+^ is 3d^5^ high spin (t_2g_^3^e_g_^2^) in O_h_ symmetry. Due to the high stability of the 3d^5^ configuration, it is difficult to observe interface charge transfer between Ni^3+^ and Fe^3+^.^32^ These properties are also found in as-prepared electrocatalysts,^17,32^ but it has remained unclear how the electronic structure evolves when applying a potential to drive electrocatalytic reactions.

Because we will analyze the spectra in terms of their valence and covalence, we introduce these concepts here. First, we define the ionic, or formal, valence and the ionic electron counts, and we subsequently define the covalent metal 3d and 4p counts based on experimental data.

A. The ionic valences are based on the stoichiometric formula of the oxides, assuming that oxygen has a formal valence of 2–, and lanthanum has a formal valence of 3+. The ionic electron counts assume that the metal 4s and 4p states are empty, and the oxygen 2p states are full. The formal valence then yields the formal 3d count, with Fe^3+^ having a 3d^5^ configuration, etc.
B. The chemical bonding between the metal 3d states and the oxygen 2p states is described within the Anderson impurity model using ligand field theory, where the parameters are derived from X-ray photoemission and X-ray absorption spectroscopy. The main effect on the chemical bonding of the 3d states is their ground-state configuration. Due to its half-filled 3d state, high-spin Fe^3+^ is stabilized and has a less covalent character to the chemical bond with oxygen as is evident from its (for a trivalent ion) high value of charge transfer parameters (Δ_eff_). Δ_eff_ can be considered as the energy distance of the O 2p state and the upper-Hubbard band of the M 3d state; it is defined with respect to the lowest multiplet levels of the 3d^4^ and 3d^5^L configurations, where L describes a ligand hole in the O 2p orbital. Fe^4+^ has a negative value of Δ_eff_ implying that 3d^5^L dominates the ground state or in other words that a large fraction of its holes are delocalized to the neighboring oxygens, implying a high covalent 3d count (difference between ionic 3d electron count and actual 3d electron count).
C. The position of transition metal K edges of binary oxides has been shown to relate linearly with the ionic valence.^33^ From this observation, we assume that the amount of metal 4p–oxygen 2p overlap increases linearly with ionic valence. In Table 1, we assume a covalent 4p count of 0.25 electrons per ionic valence unit, where we note that the exact number is not important for the present purpose, only that the covalent 4p count is linear in ionic valence.
D. The chemical bonding effects of the 3d states together with the 4p states yield the total covalency. We define the total covalency from the difference in occupation of the oxygen 2p states between the ionic limit and the actual/observed state, for example, −0.7 electron for Fe^2+^.

Table 1 shows an estimation of the iron 3d, iron 4p, and oxygen 2p occupations as a function of ionic valence, based on the assumptions A to D. The charge transfer parameters (Δ_eff_) for exemplary iron oxides are 7.8 eV for FeO, 5.3 eV for LaFeO_3_, and −3.1 eV for SrFeO_3_.^34−36^

**Table 1:** Iron–Oxygen Bond in Electron Occupations Based on XAS and XPS Spectra

	Fe 3d		Fe 4s/4p		O 2p	
Fe^2+^	6	+0.2	0	+0.50	0	–0.7
Fe^3+^	5	+0.3	0	+0.75	0	–0.7
Fe^4+^	4	+1.0	0	+1.00	0	–1.0

Here, we use in situ K-edge XAS to probe the changes of the electronic structure of LaFeO_3_ and LaFeO_3_/LaNiO_3_ thin films with 8 unit cell (u.c.) thickness during OER electrocatalysis in 1.0 M KOH solution to determine how the surface and sub-surface layers react to the OER conditions. Based on the discussion above, we expect that positive potentials will increase the ionic valence, where the 4p occupation will follow directly but the 3d occupation will not change much between Fe^3+^ and Fe^4+^. The result is that the Fe K-edge will shift to higher energy, while the pre-edge will not visibly change in position. The white line measures the lowest empty density of p-states, which implies that due to the increased metal covalent 4p count, the intensity of the white line will decrease in intensity.

## Methods

### Material Fabrications and Characterizations

**Scheme 1:**
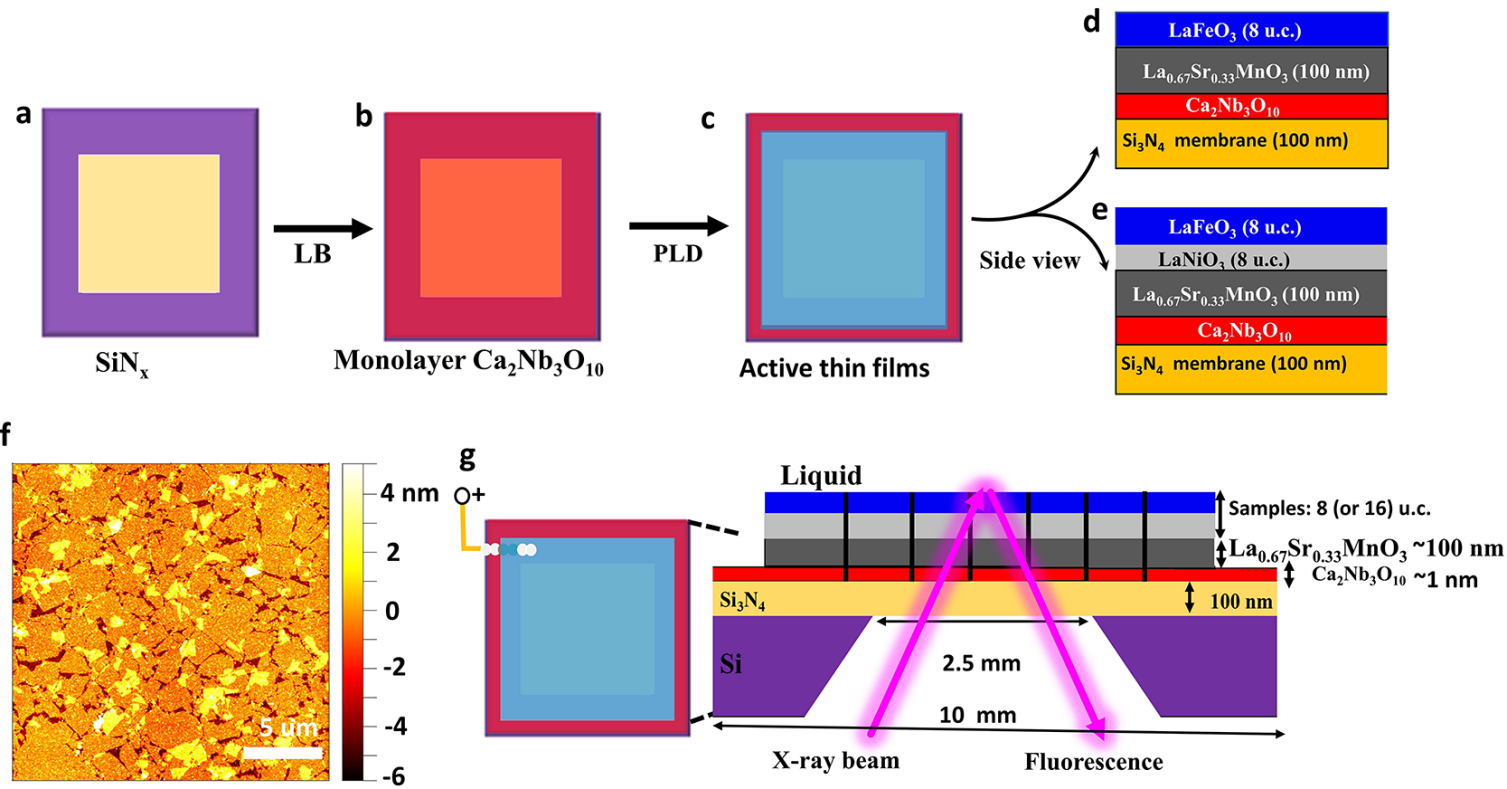
(a, b, c) The Fabrication Process of Active LaFeO_3_ and LaFeO_3_/LaNiO_3_ Thin Films with 8 u.c. Thickness via Langmuir–Blodgett (LB) and Following the PLD Process; (d) Side View: Active 8 u.c. LaFeO_3_ Thin Films; (e) Side View: Active 8/8 u.c. LaFeO_3_/LaNiO_3_ Thin Films; (f) The AFM Image of Monolayer Ca_2_Nb_3_O_10_ Nanosheets on the SiN_*x*_ Membrane; (g) The Electrode Preparation by using a Conductive Copper Wire in Contact with Silver Paste for In situ 1s XAS in 1.0 KOH Alkaline Liquid

To enable in situ XAS characterization, we employed silicon nitride membranes (SiN_*x*_), which have negligible X-ray absorption in the hard-X-ray regime. To achieve high-quality thin-film electrocatalyst layers as model systems with single out-of-plane crystal orientation, we used the recently developed approach of locally epitaxial growth of oxide thin films on arbitrary substrates using two-dimensional nanosheets (Scheme 1).^37^ The SiN_*x*_ membranes were purchased from Silson Ltd. (Southam, UK) with a frame size of 10 mm × 10 mm × 525 μm and the center membrane size of 2.5 mm × 2.5 mm x 100 nm (Scheme 1a). Monolayer Ca_2_Nb_3_O_10_ (CNO) nanosheets with a thickness of ∼1 nm were deposited on SiN_*x*_ substrates (Scheme 1b) by the Langmuir–Blodgett method, as described elsewhere.^37^^37^ Subsequently, a 100 nm conductive La_0.67_Sr_0.33_MnO_3_ (LSMO) layer serving as the electron transport layer was grown on the CNO-nanosheet buffered SiN_*x*_ substrate using the polycrystalline target via pulsed laser deposition (PLD) with a KrF excimer laser source at 248 nm. Electrocatalytically active thin films of the 8 u.c. LaFeO_3_ (Scheme 1^1^d) and 8/8 u.c. LaFeO_3_/LaNiO_3_ (Scheme 1^1^e, note: the top layer is LaFeO_3_) were epitaxially grown on the La_0.67_Sr_0.33_MnO_3_ layer. The PLD growth conditions are as follows: 1) the deposition temperature is 800 °C, 700 °C, and 500 °C, respectively, to grow 100 nm La_0.67_Sr_0.33_MnO_3_, 8 u.c. LaFeO_3_, and 8 u.c. LaNiO_3_ with oxygen partial pressures () of 0.266 mbar, 0.01 mbar, and 0.04 mbar; 2) the frequency is, respectively, 5, 2, and 2 Hz, with a spot size of 2.2 mm2, target-to-substrate distance of 50 mm and a laser fluence of 1.9 J cm^–2^ for all films; 3) comparably slow heating and cooling rates of 8 ^°^C min^–1^ under the same deposition pressure are used to avoid membrane rupture. Reference samples were produced using the same growth conditions on substrates of 100 nm SiN_*x*_ on Si (1 0 0) support.

The electrochemical performance of the samples was measured in a 3-electrode geometry in a rotating disk electrode (RDE) setup to provide a well-defined reference point for the electrochemical properties obtained in the in situ cell. To ensure electrical contact of the sample in the RDE setup, the back side, side walls, and the edges on the front side of the samples were coated with a 50 nm Pt layer by sputtering. The sample was placed at the tip of the rotary shaft using a custom-made PEEK adapter with an O-ring (FFKM, ERIKS, Germany) with 0.75 cm diameter, as described elsewhere.^17^ A BioLogic SP-300 potentiostat (Bio-Logic Science Instruments, France), a Pt wire counter electrode, and a Hg/HgO reference electrode (C3 Prozess- und Analysentechnik, Germany) were used. All potentials were converted to reversible hydrogen electrode (RHE) based on the formula: *E*_RHE_ = *E*_vs.Hg/HgO_ + 0.923. VThe samples were immersed in 1 M KOH solution prepared by dissolving KOH pellets (Sigma-Aldrich, 99.99%) in deionized water (Milli-Q, ∼18.2 MΩ cm). All RDE experiments were conducted with a rotational rate of 1600 rpm. The third cycle is shown for each sample. We found that the most severe differences occurred between the first and the second cycles, after which the CV data are more stable.

Atomic force microscopy (AFM, Bruker Dimension Icon) was used to m the seasure rface morphology, and data were analyzed using the Gwyddion software package.^38^Scheme 1^1^f presents the monolayer nanosheets of as-deposited CNO on SiN_*x*_ membranes. Epitaxy of the La_0.67_Sr_0.33_MnO_3_ layer was confirmed by using X-ray diffraction (XRD) (Figure S1).

### In Situ XAS Setup

We built a simple, low-cost home-made in situ reactor for XAS measurements by equipping a polyethylene bottle^39^ (60 mL) with three electrodes and filled it with 1.0 M KOH electrolyte. The in situ study of thin-film catalysts submerged in electrolytes is complicated by the strong absorption of X-rays in liquids, such as water. To circumvent this issue, we support our active electrocatalysts on thin SiN_*x*_ membranes that are highly transparent to hard X-rays and allow for XAS analysis from the back side. The membrane samples served as the working electrode, contacted via a copper wire using silver paste (Leitsilber, Hinkel Elektronik, Pirmasens-Winzeln, Germany). Both wire and silver paste were covered by a nonconductive epoxy-minute-adhesive glue (Weicon, Münster, Germany) to avoid any exposure of silver and copper to the electrolyte (Scheme 1g). The as-prepared electrode was mounted on the reactor hole (∼6.5 mm × 6.5 mm) by using nonconductive glue for further measurements. The saturated KCl Ag/AgCl (Metrohm, Switzerland) and a Pt wire acted as the reference electrode and counter electrode, respectively. Ex situ XAS measurements were performed without a liquid. Prior to the in situ XAS measurements, cyclic voltammetry (CV) scans from 1.2 to 1.8 V vs RHE with 10 mV s^–1^ scan rate were conducted in the static electrolyte using a potentiostat (BioLogic SP-300,France). During the XAS measurements, the potentials of the chronoamperometry measurements were set in steps from 1.2 to 2.1 V vs RHE. The potential was increased with a ramp rate of 10 mV s^–1^ between each XAS measurement, and the potential was held constant before XAS until a steady-state situation was reached, resulting in potential holds of approximately 2–3 h. Applied potentials were converted to RHE based on the formula: *E*_RHE_ = *E*_vs.Ag/AgCl_ + 0.994 V.^40^

### In Situ XAS Measurements

In situ XAS measurements were carried out on the SuperXAS beamline at the Swiss Light Source (SLS, Villigen, Switzerland), which operates at 2.4 GeV and 400 mA. A Si-coated mirror at 2.8 mrad was used for collimation and to suppress harmonic contributions. A Si (311) channel-cut monochromator was employed to measure the Fe K-edge and Ni K-edge for achieving better energy resolution. A spot size of 500 μm × 200 μm was used, with a flux of 5 × 10^10^ photons/s. For Ni K-edge, we used dwell times per point of 5 s from 8229.1 eV to 8324, 50 s from 8324 eV to 8337.5, and 5 s from 8337.5 to 8626.6 eV, while for the Fe K-edge, they were 5 s from 7011.2 to 7105.9 eV, 50 s from 7105.9 to 7119.3 eV, and 5 s from 7119.3 to 7407.6 eV. The XAS spectra were collected in FY mode, by using a 5 element silicon drift detector for the fluorescence signal and a 15 cm long ionization chamber filled with 50% He and 50% N_2_ for measuring the *I*_0_ signal. For measuring the Fe K-edge, a Mn filter was used to improve the signal/background ratio. The incident X-ray beam illuminates the active thin films from the back side (through the SiN_*x*_ membrane) under an angle of ∼45°, and the fluorescent signal is collected at a scattering angle of 90°. The XAS data analysis is described in the Supporting Information.

## Results

**Figure 1:**
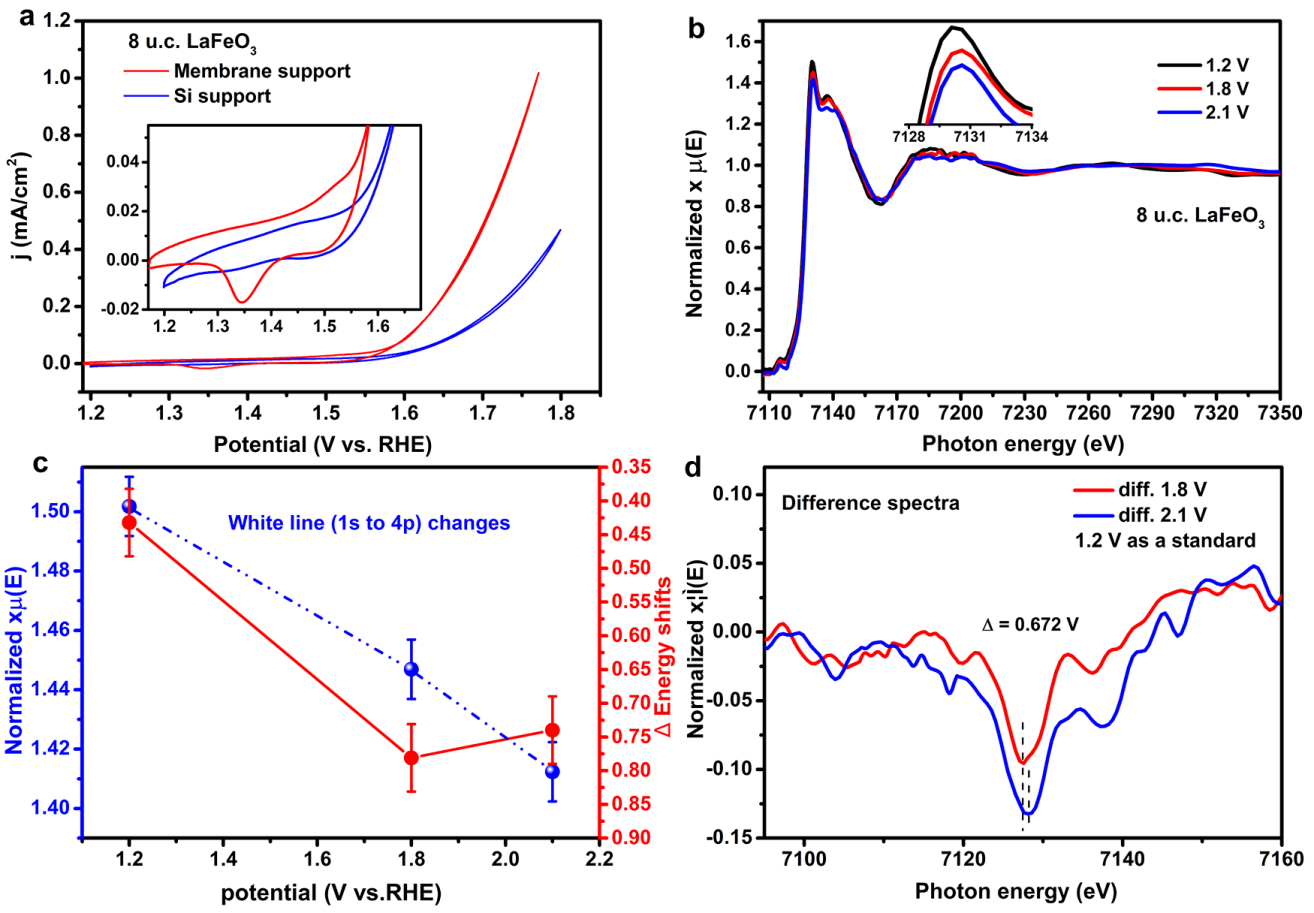
(a) CVs of 8 u.c. LaFeO_3_ thin films on membrane support and Si support. (b) In situ Fe K-edge XANES under working conditions; the inset shows the white line region enlarged. (c) The white lines (highest intensity) and energy shifts (7130 eV + Δ) as a function of potentials. (d) The related difference spectra with the 1.2 V curve.

Figure 1a shows the electrocatalytic activity of the 8 u.c. LaFeO_3_ thin films on SiN_*x*_, measured both in a standard RDE configuration and on a membrane in the in situ cell. The third identical sweep of the cyclic voltammetry data is shown (a comparison of the first and third sweeps is provided in Figure S3). Both configurations show appreciable and similar OER activity with an onset potential of 1.61 and 1.67 V vs RHE (at 100 μA/cm^2^, for the average of the cathodic and anodic sweeps, as defined by ref. (41)), confirming that the in situ cell is suitable for tracking changes under reaction conditions. The inset shows the redox features below the OER potential more clearly. Here, a small oxidation peak for the sample on the Si support can be observed around 1.42 V vs RHE, and a reduction peak is visible at 1.30 V vs RHE. The oxidation peak is partially buried in the oxygen evolution reaction current. In the in situ cell, we find the oxidation and reduction peaks at 1.52 V vs RHE and 1.35 V vs RHE, respectively. The difference in halfway potential (1.36 and 1.43 V vs RHE) may result from sample-to-sample variation or small changes in reference electrode potential between calibration in the lab and the synchrotron experiment. The larger peak separation observed for the in situ cell may result from the larger series resistance and poorer electrical contact of the sample in the in situ cell.

For the discussion of in situ XAS data, we refer to the oxidation potentials measured in the in situ cell during the XAS measurement. Figure 1b shows the in situ Fe K-edge XAS at potentials of 1.2 V vs RHE, 1.8 V vs RHE, and 2.1 V vs RHE. The extended X-ray absorption fine structure could not be detected with enough statistics due to the very thin films. We therefore focus on the X-ray absorption near-edge structure (XANES) features for the interpretation of the Fe electronic structure. As a function of the potentials, we observed three phenomena:

(1) the white line shifts to higher energy
(2) the white line loses intensity
(3) there is no detectable shift of the pre-edge position.

We quantify the energy shift by measuring the shift of the white line, taking the average of the energies of the two points where the white line reaches 95% of its maximum intensity. The exact procedure is given in the Supporting Information. Determining the energy shift in this way is more precise than determining the edge shift. We quantify the intensity of the white line by integrating the intensity using a three-point smoothing method and choose the center point between 7129.3 and 7130.9 eV. There is some apparent intensity variation in the pre-edge, but we assign this to noise/data quality; in the difference spectra in Figure 1d, no significant changes in the pre-edge region are visible. The inset in Figure 1b shows the intensity variation of the white line, i.e., the dipole 1s to 4p transition; this depends on the normalization, but that creates only a small uncertainty. The results of these quantifications are listed in Figure 1c. The procedure to determine the error bars for the energy shift and the intensity is described in the Supporting Information. The energy shift and the white line intensity both decrease approximately linearly with potential. Before discussing these results in more detail, we first show the data for the LaFeO_3_/LaNiO_3_ sample.

**Figure 2:**
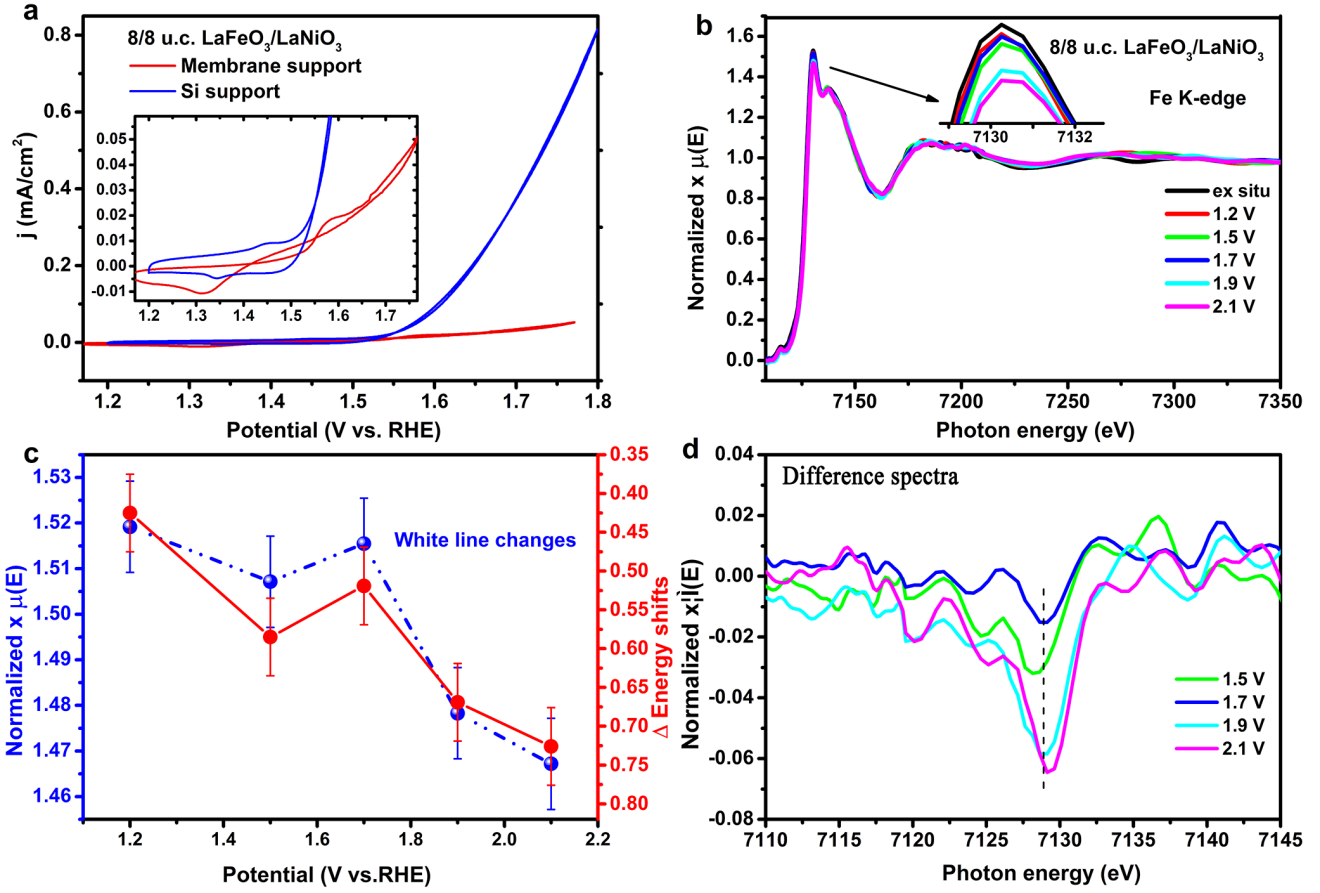
(a) CVs of LaFeO_3_/LaNiO_3_ thin films (both 8 u.c.) on both membrane and Si supports. (b) The in situ Fe K-edge XANES ex situ without liquid; the inset shows the white line region enlarged. (c) The white line (highest intensity) and energy shifts (7130 eV + Δ) as a function of potentials. (d) The corresponding difference spectra subtracted from the 1.2 V spectrum.

In Figure 2a, we report the cyclic voltammetry of the bilayer 8/8 u.c. LaFeO_3_/LaNiO_3_ thin films on a Si support measured in a standard RDE configuration and on a membrane in the in situ cell. An oxidation peak and a reduction peak are visible for the Si support sample at 1.45 vs RHE and 1.33 V vs RHE, respectively. These may be related to the Ni^2+^/Ni^3+^ redox couple.^17^ Since there are 8 u.c. LaFeO_3_ on top of the LaNiO_3_ layer, the occurrence of this redox couple implies that oxidation and reduction occur through the thin capping layer or that the growth on nanosheets results in grain boundaries where the Ni is exposed to the electrolyte. For the LaFeO_3_/LaNiO_3_ bilayer sample measured in the in situ cell, there is some crossing in the anodic and cathodic sweeps and a low OER current. We suspect that these features result from poor electrical contacting of the film in the in situ setup, which is apparent from the almost linear shape of the current density at OER potentials. Nevertheless, it is found that the possible Ni^2+^/Ni^3+^ redox feature is still present based on the reduction peak at 1.31 V vs RHE. The corresponding oxidation peak may be buried by the additional redox feature appearing at 1.45 V vs RHE. For the discussion of in situ XAS, we refer to the oxidation potentials measured in the in situ reactor. After 3.5 h of chronoamperometry during in situ XAS, the same redox features are present but more pronounced, with one additional feature at 1.68 V vs RHE (Figure S4b), suggesting a changing surface and increasing resistance. Again, these changes are likely a result of beam damage, because similar CA measurements in the RDE cell did not lead to the more pronounced or additional redox features.

Figure 2b shows the Fe K-edge XANES of the bilayer 8/8 u.c. LaFeO_3_/LaNiO_3_ thin films at in situ different potentials. The energy position and white line intensity fluctuate between 1.2 and 1.7 V. Above 1.7 V, the white line intensity decreases (Figure 2c) and the energy shifts to higher energy. Quantitatively, both the white line intensity and the energy position are identical in both the LaFeO_3_ and LaFeO_3_/LaNiO_3_ thin films. The exact numbers are given in Table S2.

**Figure 3:**
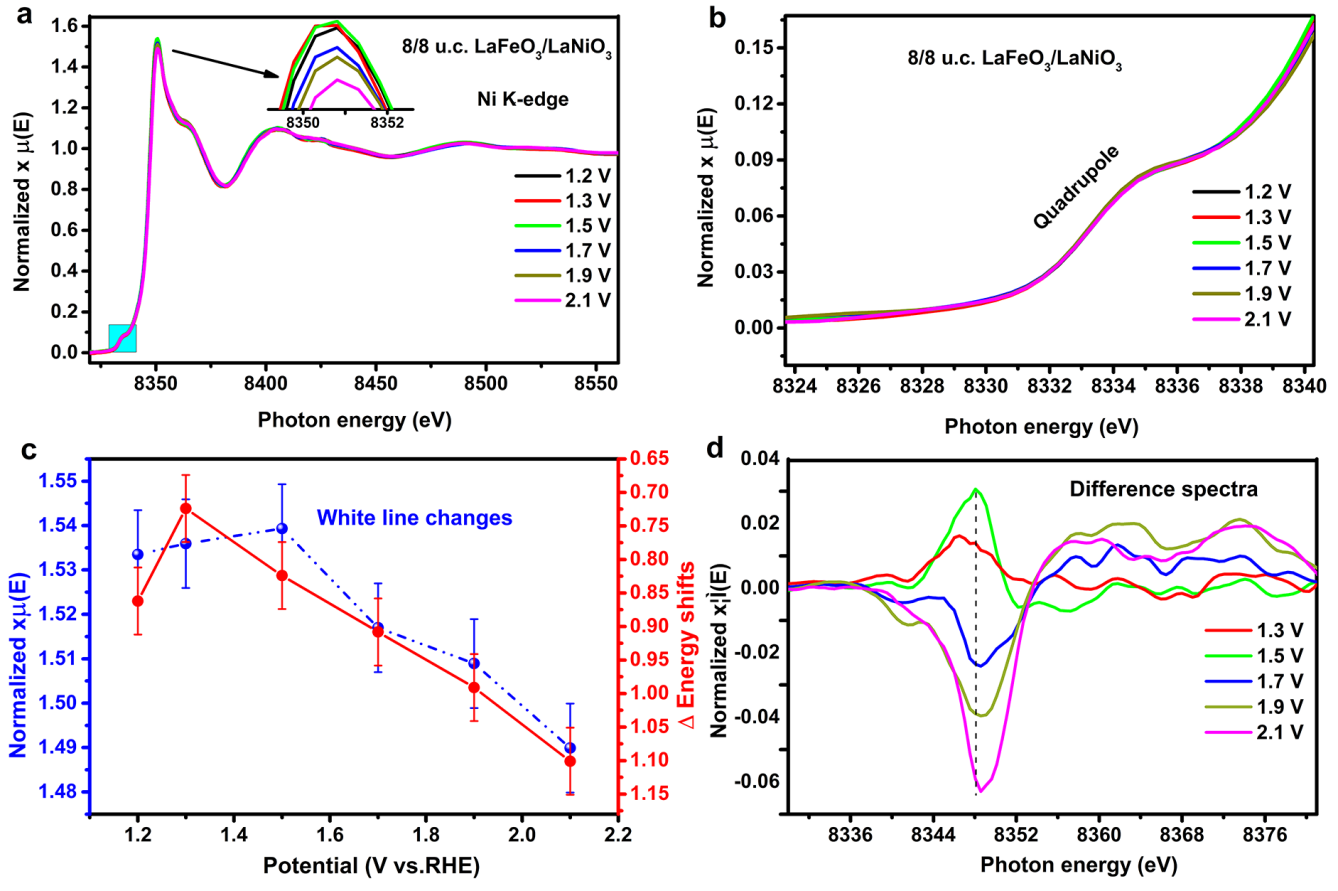
(a) The in situ Ni K-edge XANES at 1.2, 1.3, 1.5, 1.7, 1.9, and 2.1 V of 8/8 u.c. LaFeO_3_/LaNiO_3_; the inset shows the white line region enlarged. (b) Ni pre-edge K-edge at region enlarged. (c) The white line (highest intensity) and energy shifts (8350 eV + Δ) as a function of potentials. (d) Corresponding difference spectra using a standard spectrum at 1.2 V.

Figure 3 shows the Ni K-edge results of the bilayers of LaFeO_3_/LaNiO_3_, where we note that the LaNiO_3_ layer is sandwiched between the LaFeO_3_ and La_0.67_Sr_0.33_MnO_3_ layers. Figure 3b shows the changes in the pre-edge shape does not change visibly in intensity and position with increasing potential from 1.2 to 2.1 V (Figure 3b). Figure 3c shows a decrease in the white line intensity, and an energy shift to higher energy for potentials above 1.5 V.

## Discussion

### Analysis of the Energy Shifts and White Line Decrease

We observe for the Fe and Ni K-edges of the LaFeO_3_/LaNiO_3_ system and for the Fe K-edge of the LaFeO_3_ system the following three trends:

(1) the edge position shifts to higher energy
(2) the white line loses intensity
(3) there is no detectable shift of the pre-edge position, (within the accuracy of the data).

Because the LaNiO_3_ is sandwiched between LaFeO_3_ and La_0.67_Sr_0.33_MnO_3_, these observations seem not related to the oxide/electrolyte interface but to the averaged reaction of the whole active oxide layer. In principle, there could be some Ni exposed to the electrolyte due to diffusion to the surface or electrolyte penetrating the grain boundaries. This will be a minor amount that cannot quantitatively explain the changes in the Ni K edge spectra, which are equivalent to the Fe K edge spectra. For the bilayer, the changes in the edge shift start to occur from 1.7 V vs RHE and 1.5 V vs RHE for the Fe K-edge and the Ni K-edge, respectively. In the cyclic voltammetry, this is just after the redox features. So the oxidation peak at ∼1.4 V vs RHE is likely related to Ni as argued before, and the oxidation peak at ∼1.6 V vs RHE could be related to some oxidation of the Fe. Table 2 compares the edge shift and the white line decrease for the three measured K edges between voltages of 1.2 and 2.1 V

**Table 2:** Comparison of the Energy Shift (Δ*E*) and White Line Intensity (Δ*I*) Changes between 1.2 and 2.1 V

thin films	absorption edges	Δ*I*	Δ*E*
8 u.c. LaFeO_3_	Fe K	–0.09	+0.31
8/8 u.c. LaFeO_3_/LaNiO_3_	Fe K	–0.05	+0.30
8/8 u.c. LaFeO_3_/LaNiO_3_	Ni K	–0.05	+0.24

### X-ray Absorption Spectra with Respect to Valence

As a rule of thumb, the valence change in transition metal K edge XAS shifts the energy of the pre-edge position by ∼1.5 eV and the white line position by ∼4 eV per valence change.^42,43^ In case of equivalent systems, the ionic valence (or formal oxidation state) correlates linearly with the pre-edge and edge position of K edge X-ray absorption (1s to 3d pre-edge quadrupole and 1s to 4p dipole).^33^ Our in situ experiment indicates that the pre-edge features for both the Ni and Fe K-edge of LaFeO_3_ and LaFeO_3_/LaNiO_3_ thin films at higher potentials do not visibly shift. We argue that due to the negative charge transfer value of both Fe^4+^ and Ni^4+^, the 3d count does not change much upon oxidation from Fe^3+^ (5.3) to Fe^4+^ (5.0) as indicated in Table 1. The consequence is that the pre-edge does not shift, while the edge shifts.

The white line intensity of the iron and nickel K edges probes the empty p projected density of states. In a transition metal oxide, the metal 4p band is the antibonding combination of the metal 4p states and the oxygen 2p states. The intensity of this peak at the iron K edge probes the Fe character of this band, and a decrease in this intensity implies a decrease in the Fe 4p character of that band. This implies an increase in the oxygen 2p character of that peak. In a fully ionic, noncovalent situation, the 4p band has no oxygen 2p character. Any increase in the oxygen 2p character of the Fe 4p band therefore implies an increased covalence between Fe 4p and the O 2p orbitals. In turn, an increased covalence implies shorter Fe–O distances. A shift of the energy position to higher energy is often interpreted as an increased valence. The reason for the edge shift is related to a change in the metal–oxygen distance. Table 2 shows that the edge shift and the white line intensity decrease are similar for the edges observed.

We conclude that the LaFeO_3_ and LaNiO_3_ perovskite layers show shorter Fe–O and Ni–O distances and increased valence and covalence as functions of the applied potential. We note again that the change in valence from Fe^3+^ to Fe^4+^ leaves the number of 3d electrons largely unaffected as summarized in Table 1. This behavior is very likely an effect of the whole 8 u.c. perovskite layer and should be distinguished from the redox behavior of the LaFeO_3_/electrolyte interface. The valence in transition metal oxides plays an important role in the activity of catalysts and battery systems. We expect the increasing valence with increasing potential to be a general trend for transition metal oxides. Applying a potential across an oxide system could modify the metal–oxygen distances and the strength of the metal–oxygen bonding.

## Conclusions

The in situ K-edge XAS of LaFeO_3_ and LaFeO_3_/LaNiO_3_ thin films with 8 u.c. thickness for the OER reaction show significant spectral changes under working conditions in 1.0 M KOH solution. The reduced Fe and Ni K-edge white lines indicate reduced metal 4p character, hence increased O 2p character, and, in other words, increased (co)valence. The increased valence is related to shorter metal 4p–O 2p bonds, which is visible in the Fe and Ni K energy shifts. Both the energy shift and the white line decrease follow the same trends for all three spectra studied. While the valence is increasing, the pre-edge positions of Ni and Fe remain constant related to the negative charge transfer energies of the 4+ ions. Because the effect is equivalent in the surface LaFeO_3_ and sub-surface LaNiO_3_ film, we conclude that the here described behavior is caused by the effect of the potential on the whole thin film perovskites, not just the surface. We expect that this effect is general for many active solid/liquid interfaces under potential conditions.

## Supporting Information Available

XRD, CVs on Si, and in situ XAS energy shifts; XRD of 8 u.c. LaNiO_3_ on 100 nm LSMO-SiN_*x*_ (Figure S1); the fabrication of active thin films on 0.5 mm thickness Si substrate using the same conditions for the membranes (Figure S2); CVs of 8 u.c. LaFeO_3_ (Figure S3) and 8/8 u.c. LaFeO_3_/LaNiO_3_ (Figure S4) thin films on membrane support; data treatment of Ni K-edge from the 8/8 u.c. LaFeO_3_/LaNiO_3_ thin films (Figure S5); the comparison of normalized [0,1] in-situ Fe K-edge XAS data at 1.2 V and 2.1 V for 8 u.c. LaFeO_3_ and 8/8 u.c. LaFeO_3_/LaNiO_3_ thin films (Figure S6); an example of reference Fe foil K-edge XAS (raw data, without normalization) is measured for both ex situ 8 u.c. LaFeO_3_ and 8/8 u.c. LaFeO_3_/LaNiO_3_ thin films (Figure S7); an example of Ni 8/8 u.c. LaFeO_3_/LaNiO_3_ at 1.2 V to determine peak intensity and position error (Figure S8); the measurement conditions of the active materials on different supports (Table S1); energy shifts at 95% of all operando XAS data normalized [0,1] (Table S2); error analysis of Ni 8/8 u.c. LaFeO_3_/LaNiO_3_ at 1.2 V (Table S3) (PDF)
