# Supplementary material for: In Situ X-ray Absorption Spectroscopy of LaFeO3 and LaFeO3/LaNiO3 Thin Films in the Electrocatalytic Oxygen Evolution Reaction
Source: J Phys Chem C Nanomater Interfaces. 2024 Mar 20;128(13):5515–23. doi: 10.1021/acs.jpcc.3c07864 (PMC11000219; doi:10.1021/acs.jpcc.3c07864)
Supplement: Supplementary file 1 — jp3c07864_si_001.pdf [file jp3c07864_si_001.pdf]

In Situ X-ray Absorption Spectroscopy of LaFeO3 and LaFeO3/LaNiO3 Thin Films in the Electrocatalytic Oxygen Evolution Reaction

Qijun Che[a], Iris C. G. van den Bosch[b], Phu T. P. Le[b], Masoud Lazemi[a], Emma van der Minne[b], Yorick A. Birkhölzer[b], Moritz Nunnenkamp[b], Matt L. J. Peerlings[a], Olga V. Safonova[c], Maarten Nachtegaal[c], Gertjan Koster[b], Christoph Baeumer[b], Petra de Jongh[a], Frank M. F. de Groot[a, *]

Address

[a] Materials Chemistry and Catalysis, Debye Institute for Nanomaterials Science, Utrecht University, Universiteitsweg 99, 3584 CG Utrecht, the Netherlands, corresponding author: Frank de Groot, *email: f.m.f.degroot@uu.nl

[b] MESA+ Institute for Nanotechnology, University of Twente, 7500 AE Enschede, The Netherlands

[c] Paul Scherrer Institute, CH-5232 Villigen, Switzerland

Tables and Figure

Table S1. The summarized measurement conditions of active materials on different support

<table>
<tr><th colspan="2">Samples</th><th>Exposed area (cm^2)</th><th colspan="2">Cell</th><th>Solutions</th></tr>
<tr><td rowspan="2">Membrane support</td><td>8 u.c. $LaFeO_3$</td><td>0.18000</td><td rowspan="2">Operando reactor Ag/AgCl</td><td rowspan="2"></td><td rowspan="2">1.0 KOH</td></tr>
<tr><td>8/8 u.c. $LaFeO_3/LaNiO_3$</td><td>0.15966</td></tr>
<tr><td rowspan="2">Si support</td><td>8 u.c. $LaFeO_3$</td><td rowspan="2">0.4418</td><td colspan="2" rowspan="2">RDE setup Hg/HgO</td><td rowspan="2">1.0 KOH</td></tr>
<tr><td>8/8 u.c. $LaFeO_3/LaNiO_3$</td></tr>
</table>

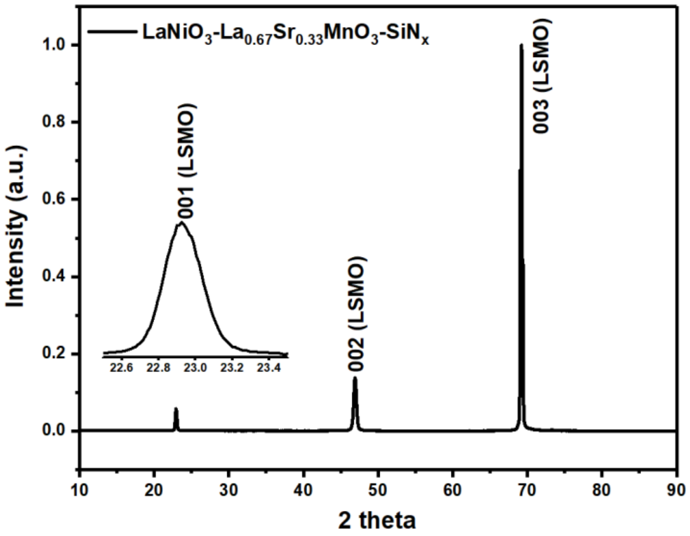

Figure S1. An example of X-ray powder diffraction (XRD) of 8 u.c. $LaNiO_3$ on 100 nm LSMO-SiN_x.

1. XRD analysis

The XRD was recorded on a Bruker D2 PHASER. The example X-ray diffraction experiments of 3.1 nm $LaNiO_3$ on $La_{0.67}Sr_{0.33}MnO_3$ -SiN_x show three $La_{0.67}Sr_{0.33}MnO_3$ peaks: (0 0 1), (0 0 2), and (0 0 3). It is not unexpected that the $LaNiO_3$ is not visible (Figure S1), because of the similar lattice constant yet small quantity compared to 100 nm $La_{0.67}Sr_{0.33}MnO_3$.

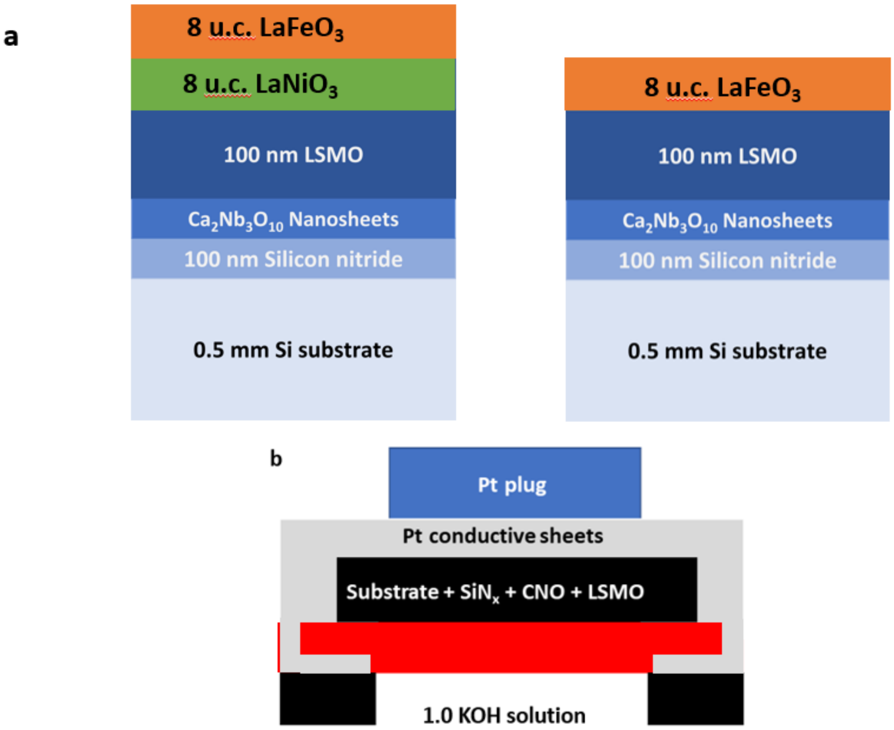

Figure S2. (a) The fabrication of active thin films on 0.5 mm thickness Si substrate using the same process conditions as on the membrane support: 8/8 u.c. $LaFeO_3/LaNiO_3$, 8 u.c. $LaFeO_3$ catalysts, (b) RDE set-up for electrochemistry measurement.

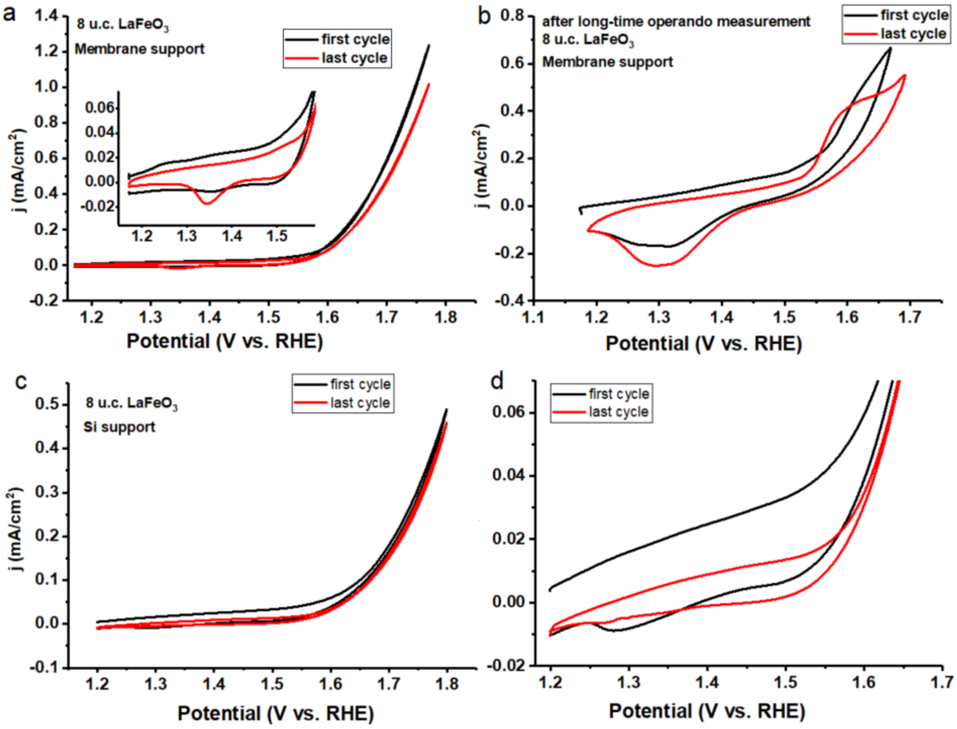

Figure S3. The CVs of 8 u.c. $LaFeO_3$ thin films on membrane support: (a) with first and third cycles, before operando XAS. Inset: zoom in at low current density to show redox peaks. (b) after

long time operando measurements with first and third cycles.

8 u.c. $LaFeO_3$ on Si support: (c) The CVs of first and third cycles, inset (d) zoom in at low current density to show redox peaks.

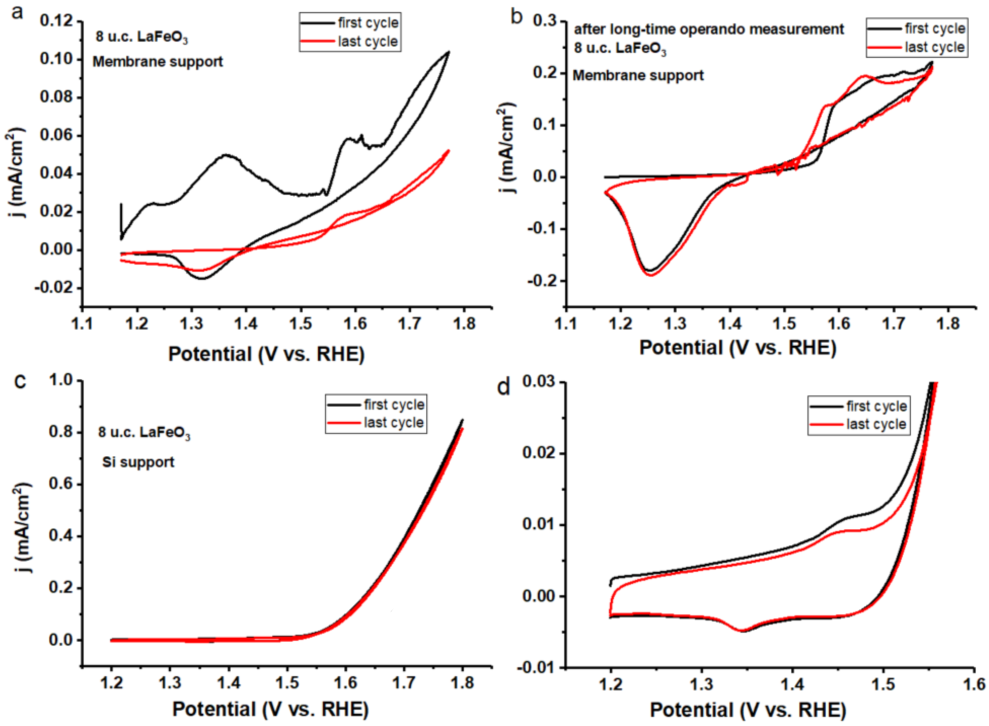

Figure S4 The CVs of 8/8 u.c. $LaFeO_3/LaNiO_3$ thin films on membrane support: (a) with first and third cycles, before operando XAS. (b) after long time operando measurements with first and third cycles. 8/8 u.c. $LaFeO_3/LaNiO_3$ on Si support: (c) The CVs of first cycle and last cycle, inset (d) zoom in at low current density to show redox peaks.

Operando XAS procedure

(1) We measured the Fe K-edge 4 times and the Ni K-edge 3 times respectively on every external potential and combined the data to obtain an average spectrum.

(2) All XAS data processes were performed with open code of Athena [1] (Version 0.9.22, https://bruceravel.github.io/demeter/).

(3) For all operando and ex-situ XAS spectra we used a three-point smoothing.

An example of the treatment of the Ni K-edge from the raw data

This example of 8/8 u.c. $LaFeO_3/LaNiO_3$ thin films shows the processing of the raw XAS data; the other XAS data follow the same process. We merged all XAS curves at the same potential and normalized the data, as shown in Figure S5; a three-point smoothing method was used; processed data is shown in the manuscript figures 1, 2, and 3.

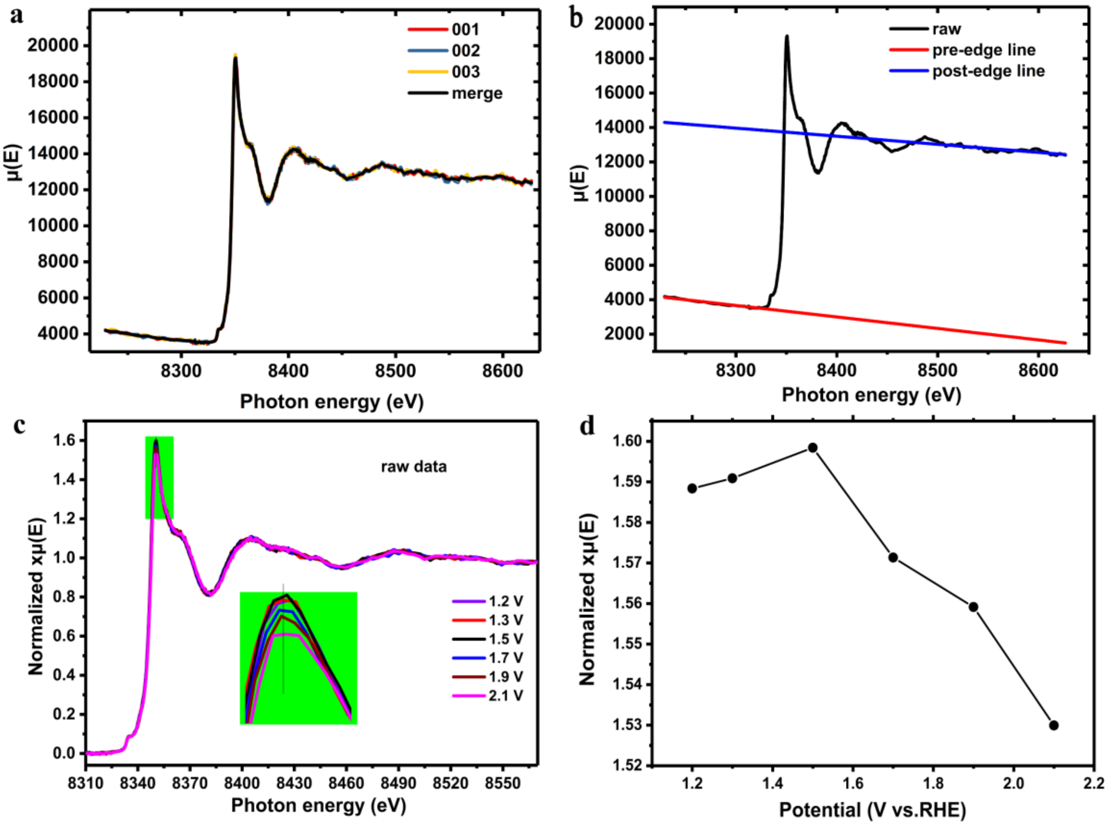

Figure S5. The data treatment of the Ni K-edge from the 8/8 u.c. $LaFeO_3/LaNiO_3$ thin films:

(a) The Ni K-edge XAS recorded repeated three times under the operando condition of 1.2 V.

(b) The merged absorption curve at 1.2 V and their pre-edge subtraction and normalization.

(c) The normalized data from 1.2 V to 2.1 V.

(d) The corresponding white line as a function of potential.

Procedure to determine the energy shift

We normalized all spectra from zero to one for the two different samples of 8 u.c. $LaFeO_3$ and 8 u.c. $LaFeO_3/LaNiO_3$ under all potentials. We determine the energy shift from 1.2 V to 2.1 V, also indicated graphically in Figure S6. We added the energies where the line crosses 0.95 (up and down) to calculate the energy shift for all operando XAS data (Table S2). These numbers are used in the manuscript.

Table S2 energy shifts at 95% of all operando XAS data normalized [0,1]

Potential / *K-edges*	ΔE (eV)[*a,b]						
	1.2 V	1.3 V	1.5 V	1.7 V	1.8 V	1.9 V	2.1 V
Fe 8 u.c. $LaFeO_3$	0.43	-	-	-	0.78	-	0.74
Fe 8/8 u.c. $LaFeO_3/LaNiO_3$	0.43		0.58	0.52		0.67	0.73
Ni 8/8 u.c. $LaFeO_3/LaNiO_3$	0.86	0.72	0.82	0.91		0.99	1.10

These values should be added to the value 7130 eV for Fe and 8350 eV for Ni. (Thus 0.43 indicates an energy of 7130.43 eV, etc.)

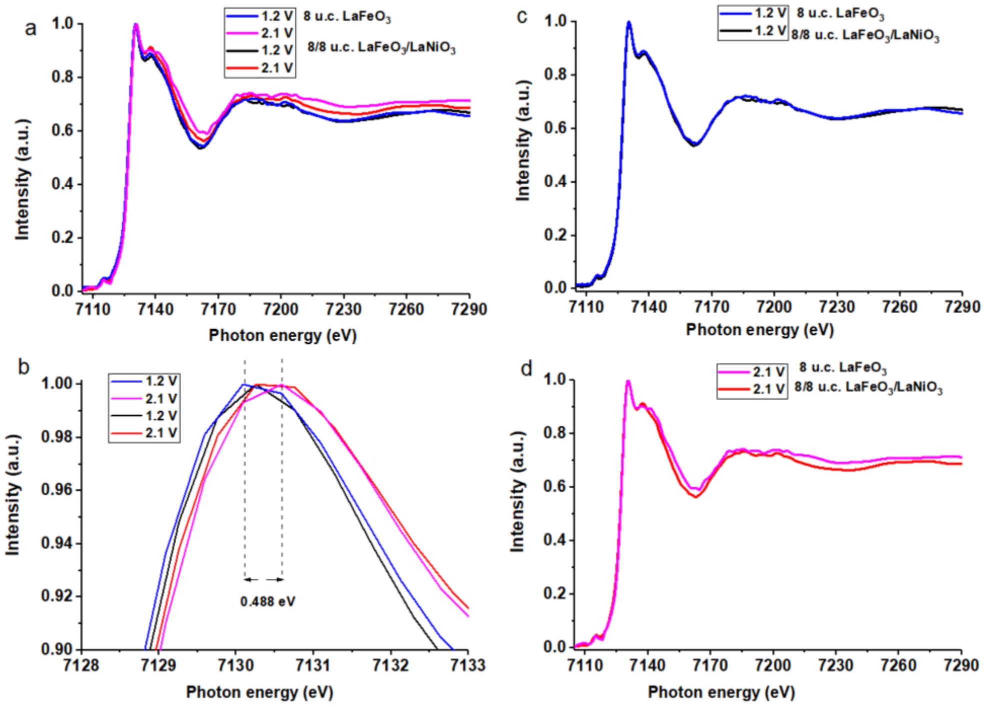

Figure S6 The comparison of normalized [0,1] operando Fe K-edge XAS data at 1.2 V and 2.1 V for 8 u.c. $LaFeO_3$ and 8/8 u.c. $LaFeO_3/LaNiO_3$ thin films.

Error analysis

Synchrotron measurement sequence:

(1) 8/8 u.c. $LaFeO_3/LaNiO_3$ thin films,

(2) 8 u.c. $LaFeO_3$ thin films.

(a) XAS peak position

- ***We used reference Fe-foil to track possible beamline energy shifts***

The reference Fe foil K-edge XAS is recorded during the measurements of both 8/8 u.c. $LaFeO_3/LaNiO_3$ and Fe K-edge of 8 u.c. $LaFeO_3$ thin films. As shown in Figure S7, there is no detectable absolute energy shift, which means that the energy calibration (peak position) error due to calibration is near zero.

- ***Data treatments for smooth from three-point method***

Due to background noise of the raw data we use a three-point smooth method to get better XAS curves to analyze; the parameters are the same in all XAS data processes, which means the whole system errors are equivalent for the two samples, implying that it will not influence the relative quantitative analysis. We give an example of the Ni K-edge XAS of 8/8 u.c. $LaFeO_3/LaNiO_3$ at 1.2 V thin films to estimate the error. We used $\mu(x)$ ranging from 0.7 to 1.2 to determine 6 points to estimate the average error and standard deviation.

Position average shifts:± 0.05 eV, standard deviation: 0.06

Intensity average shifts:± 0.003 eV(in the manuscript, we use 3.33 times and that is 0.01), standard deviation: $= 0.009$

Table S3 Error analysis of Ni 8/8 u.c. $LaFeO_3/LaNiO_3$ at 1.2 V from raw to smoothed data

<table>
<tr><td>$\mu(x)$</td><td>0.7</td><td>0.8</td><td>0.9</td><td>1.0</td><td>1.1</td><td>1.2</td></tr>
<tr><td>Position row data (eV)</td><td>8346.367</td><td>8346.782</td><td>8347.170</td><td>8347.530</td><td>8347.862</td><td>8348.167</td></tr>
<tr><td>Position smoothed (eV)</td><td>8346.256</td><td>8346.699</td><td>8347.087</td><td>8347.474</td><td>8347.862</td><td>8348.250</td></tr>
<tr><td>Position shift (eV)</td><td>-0.111</td><td>-0.083</td><td>-0.083</td><td>-0.056</td><td>0</td><td>0.083</td></tr>
<tr><td colspan="7">Position error
Average shifts:
$\left|\frac{\sum -0.111-0.083-0.083-0.056+0-0.083}{6}\right| = 0.05$ eV;
Position standard deviation:
$\delta = \sqrt{((-0.111+0.05)^2 + 2*(-0.083+0.05)^2 + (-0.056+0.05)^2 + 0.05^2 + (0.083+0.05)^2)/6} = 0.066$</td></tr>
<tr><td>Energy (eV)</td><td>8346.3</td><td>8346.7</td><td>8347.1</td><td>8347.5</td><td>8347.9</td><td>8348.3</td></tr>
<tr><td>Intensity row data</td><td>0.687</td><td>0.784</td><td>0.887</td><td>0.998</td><td>1.115</td><td>1.229</td></tr>
<tr><td>Intensity smoothed</td><td>0.702</td><td>0.793</td><td>0.894</td><td>0.999</td><td>1.106</td><td>1.222</td></tr>
<tr><td>Intensity shifts</td><td>0.015</td><td>0.009</td><td>0.007</td><td>0.001</td><td>-0.009</td><td>-0.007</td></tr>
<tr><td colspan="7">Intensity error
average shifts: $\left|\frac{\sum 0.015+0.009+0.007+0.001-0.009-0.007}{6}\right|$ =0.00267;
standard deviation: $\delta = \sqrt{(x_i - 0.00267)^2/6}$=0.0086, x_i is intensity shifts.</td></tr>
</table>

- ***Energy shifts determination from points at 95%***

  In Table S2 we use all smoothed XAS data normalized from zero to one. Based on the estimated error from Table S3, we estimate the error of the energy shifts as $\pm$0.05 eV at 95 %, and use this value in the manuscript figures 1c, 2c ,3c. We note that from Table S2, for the two independent samples, the Fe K-edges energy shift difference is 0.01 eV from 1.2 V to 2.1 V, which indicate that the energy shifts determination from points at 95% for energy shift error is 0.01.

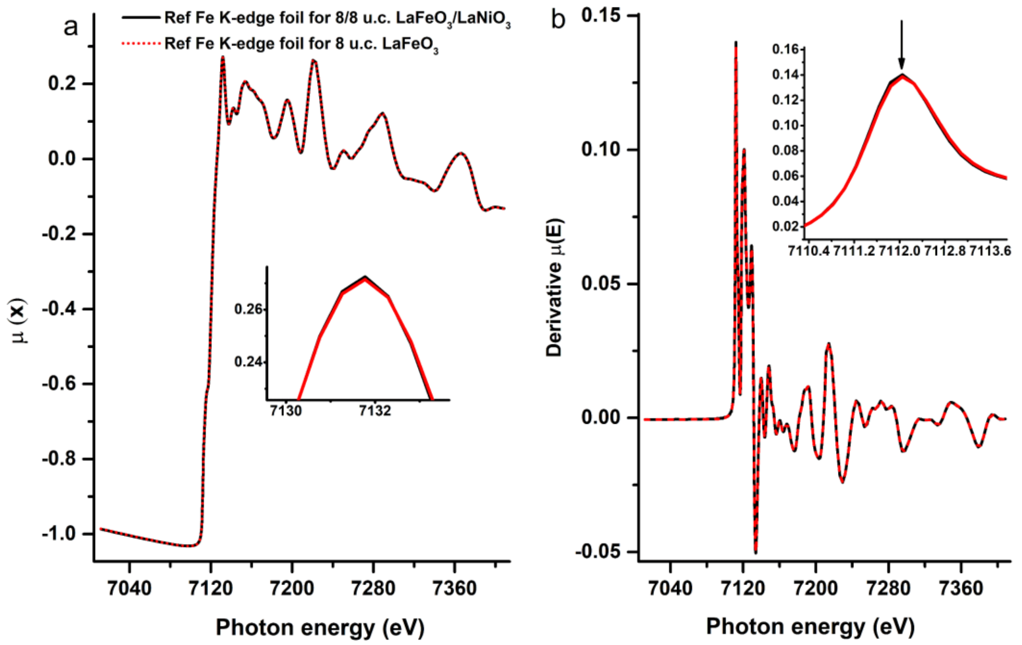

Figure S7 (a) An example of reference Fe foil K-edge XAS (raw data, without normalization) is measured for both ex-situ 8 u.c. $LaFeO_3$ and 8/8 u.c. $LaFeO_3/LaNiO_3$ thin films, insert enlarged white line. (b) Related first derivative from (a), insert the sharpest peak.

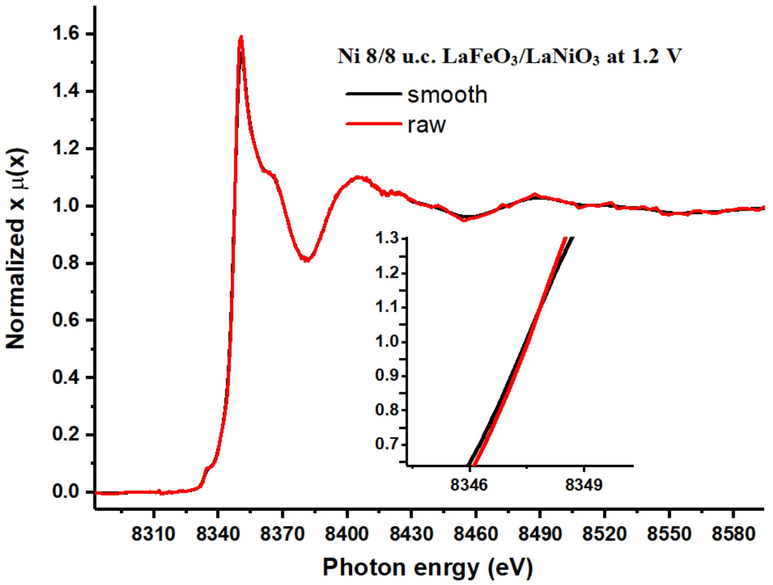

Figure S8 An example of Ni 8/8 u.c. $LaFeO_3/LaNiO_3$ at 1.2 V to determine peak intensity and position error, the value see Table S3.

References

[1] B. Ravel and M. Newville, ATHENA, ARTEMIS, HEPHAESTUS: data analysis for X-ray absorption spectroscopy using IFEFFIT, Journal of Synchrotron Radiation 12, 537-541 (2005).
